# Air Pollution–Mediated Susceptibility to Inflammation and Insulin Resistance: Influence of CCR2 Pathways in Mice

**DOI:** 10.1289/ehp.1306841

**Published:** 2013-10-22

**Authors:** Cuiqing Liu, Xiaohua Xu, Yuntao Bai, Tse-Yao Wang, Xiaoquan Rao, Aixia Wang, Lixian Sun, Zhekang Ying, Liubov Gushchina, Andrei Maiseyeu, Masako Morishita, Qinghua Sun, Jack R. Harkema, Sanjay Rajagopalan

**Affiliations:** 1 Department of Physiology, Hangzhou Normal University, Hangzhou, China; 2 The Ohio State University, Columbus, Ohio, USA; 3 Division of Cardiovascular Medicine, University of Maryland, Baltimore, Maryland, USA; 4 Environmental Health Sciences, University of Michigan, Ann Arbor, Michigan, USA; 5 Department of Pathobiology and Diagnostic Investigation, Michigan State University, Lansing, Michigan, USA

## Abstract

Background: Epidemiologic and experimental studies support an association between PM_2.5_ exposure and insulin resistance (IR). Innate immune cell activation has been suggested to play a role in the pathogenesis of these effects.

Objectives: We sought to evaluate the role of CC-chemokine receptor 2 (CCR2) in PM_2.5_-mediated inflammation and IR.

Methods: Wild-type C57BL/6 and CCR2^–/–^ male mice were fed a high-fat diet and exposed to either concentrated ambient PM_2.5_ or filtered air for 17 weeks via a whole-body exposure system. We evaluated glucose tolerance and insulin sensitivity. At euthanasia, blood, spleen, and visceral adipose tissue (VAT) were collected, and inflammatory cells were measured using flow cytometry. We used standard immunoblots, immunohistochemical methods, and quantitative PCR (polymerase chain reaction) to assess pathways of interest involving insulin signaling, inflammation, and lipid and glucose metabolism in various organs. Vascular function was assessed using myography.

Results: PM_2.5_ exposure resulted in whole-body IR and increased hepatic lipid accumulation in the liver, which was attenuated in CCR2^–/–^ mice by inhibiting SREBP1c-mediated transcriptional programming, decreasing fatty acid uptake, and suppressing p38 MAPK activity. Abnormal phosphorylation levels of AKT, AMPK in VAT, and adipose tissue macrophage content in wild-type mice were not present in CCR2^–/–^ mice. However, the impaired whole-body glucose tolerance and reduced GLUT-4 in skeletal muscle in response to PM_2.5_ was not corrected by CCR2 deficiency.

Conclusions: PM_2.5_ mediates IR by regulating VAT inflammation, hepatic lipid metabolism, and glucose utilization in skeletal muscle via both CCR2-dependent and -independent pathways. These findings provide new mechanistic links between air pollution and metabolic abnormalities underlying IR.

Citation: Liu C, Xu X, Bai Y, Wang TY, Rao X, Wang A, Sun L, Ying Z, Gushchina L, Maiseyeu A, Morishita M, Sun Q, Harkema JR, Rajagopalan S. 2014. Air pollution–mediated susceptibility to inflammation and insulin resistance: influence of CCR2 pathways in mice. Environ Health Perspect 122:17–26; http://dx.doi.org/10.1289/ehp.1306841

## Introduction

Recent epidemiologic studies and experimental evidence support adverse cardiometabolic consequences of air-pollution exposure by worsening of whole-body insulin sensitivity (Rajagopalan and Brook 2012). Studies from our group first demonstrated that exposure to PM_2.5_ (particulate matter < 2.5 μm) exaggerates insulin resistance (IR) and visceral inflammation/adiposity in mice fed either a high-fat diet (HFD) or a normal diet (Sun et al. 2009; Xu et al. 2010). Inflammation in insulin-sensitive tissues, such as visceral adipose tissue (VAT) and liver, is a central abnormality in obesity/insulin resistance (IR) (Hotamisligil 2006; Ouchi et al. 2011; Shoelson et al. 2006), with recruitment of innate immune cells (e.g., monocytes) into adipose tissue and the liver driving the development of glucose and lipoprotein dysregulation (Lumeng et al. 2008; Weisberg et al. 2003, 2006; Xu et al. 2003).

CC-chemokine receptor 2 (CCR2) plays a critical role in the entry of innate immune cells into tissue through direct interaction with its ligands, CCL2 (monocyte chemoattractant protein 1; MCP-1), CCL7, CCL8, and CCL12 (Charo and Ransohoff 2006; Proudfoot 2002). Recent studies have shown that the CCR2/CCL2 system is not only critical to VAT inflammation but also to the recruitment of macrophages to the liver in response to an HFD (Oh et al. 2012). Consistent with a central role in immune cell recruitment, CCR2 deficiency ameliorates obesity, VAT inflammation, and systemic IR; in fact, hematopoietic CCR2 deficiency is essential for improvement (Ito et al. 2008; Weisberg et al. 2006). In light of the obligatory role of the innate immune system in PM_2.5_ effects and data presented in the studies cited above, we hypothesized that the adverse effects of PM_2.5_ exposure on metabolic dysregulation are mediated through coordinated effects on the liver and VAT. We systematically investigated this question in wild-type (WT) and CCR2^–/–^ mice subjected to air pollution exposure.

## Materials and Methods

*Animals.* Male C57BL/6 WT and CCR2^–/–^ mice were purchased from Jackson Laboratories (Bar Harbor, ME, USA). At 18 weeks of age, all mice were housed in groups and maintained at 21°C on a 12-hr light/12-hr dark cycle; they had free access to water and were fed an HFD that derived 60% of calories from lipids (Harlan Teklad, Indianapolis, IN, USA). The protocols and the use of animals were approved by and in accordance with The Ohio State University Animal Care and Use Committee, and the animals were treated humanely and with regard for alleviation of suffering. To avoid sex-dependent differences, we included only male mice in the study.

*Whole-body inhalation.* Both WT and CCR2^–/–^ (CCR2) mice were exposed by inhalation to either filtered air (FA) or concentrated PM_2.5_ (PM) for 6 hr/day, 5 days/week from 28 November 2011 to 23 March 2012 (a total duration of 117 days; ~ 17 weeks). Inhalation exposure was carried out in a mobile exposure system, the Ohio Air Pollution Exposure System for Interrogation of Systemic Effects, located at The Ohio State University Animal Facility (Columbus, OH, USA). The animal groups were as follows: WT-FA (*n* = 8), WT-PM (*n* = 9), CCR2-FA (*n* = 9), and CCR2-PM (*n* = 8). Animal exposures and monitoring of the exposure environment were performed as described previously (Sun et al. 2009; Xu et al. 2010).

*PM_2.5_ concentration and composition in the exposure chambers.* To calculate PM_2.5_ mass concentrations in the exposure chambers, samples were collected on Teflon filters (Teflo; 37 mm, 2-μm pore; PALL Life Sciences, Ann Arbor, MI, USA) at least once each week. Gravimetric determinations were made using an MT-5 microbalance (Mettler Toledo, Columbus, OH, USA) in a temperature- and humidity-controlled Class 100 clean laboratory. PM samples collected on Teflon filters were wet with ethanol and extracted in 10% nitric acid solution. Sample extracts were then analyzed for a suite of trace elements using inductively coupled plasma-mass spectrometry (ELEMENT2; Thermo Finnigan, San Jose, CA, USA) as described by Morishita et al. (2011).

*Blood glucose homeostasis and insulin sensitivity.* Four days before exposure (baseline) and 4 days after inhalation exposure ended, mice were fasted overnight, and blood samples were collected for assessment of fasting insulin/glucose levels. Glucose tolerance was then analyzed using an intraperitoneal (ip) glucose tolerance test (IPGTT). For this test, mice were administered glucose (2 mg/​kg) by ip injection and blood was collected at 0, 30, 60, 90, and 120 min for blood glucose measurement.

One day before exposure to FA or PM_2.5_, after 8 weeks of exposure, and again 1 day after the exposure ended, mice were fasted for 4.5 hr and assessed for insulin sensitivity by the insulin tolerance test (ITT) (Hagiwara et al. 2012). For this test, mice were administered insulin (0.5 U/kg) by ip injection and blood was collected at 0, 30, 60, 90, and 120 min for blood glucose measurement.

We then performed homeostasis model assessment of the IR index (HOMA-IR), calculated based on 1 mg of insulin being equivalent to 24 IU, using the formula HOMA-IR = [fasting insulin concentration (nanograms per milliliter) × 24 × fasting glucose concentration (milligrams per deciliter)] ÷ 405 (Xu et al. 2010). The HOMA of β-cell function (HOMA-β) was calculated using the formula HOMA-β = 360 × [fasting insulin concentration (nanograms per milliliter)] ÷ [fasting glucose concentration (milligrams per deciliter)] – 63, with values given as percentages.

*Circulating inflammatory biomarkers and lipid profile.* Cytokine levels in plasma were determined using a Cytometric Bead Array (BD Biosciences, San Diego, CA, USA). Serum was incubated with beads specific for tumor necrosis factor α (TNFα), interferon γ (IFNγ), monocyte chemoattractant protein 1 (MCP-1), interleukin (IL)-6, IL-10, and IL-12p70 according to the manufacturer’s instructions. The total amount of cytokines was determined using a BD LSR II flow cytometer and analyzed by BD CBA software (BD Biosciences) (Xu et al. 2011). Triglyceride and cholesterol levels in liver and/or blood were measured according to the methods of Allain et al. (1974) and McGowan et al. (1983).

*Myograph study.* After the final IPGTT and ITT measurements, which took place at the end of 17 weeks of PM_2.5_ or FA exposure, mice were killed by isoflurane inhalation. The thoracic aorta, with adhesive tissue removed, was dissected out, and vascular function (acetylcholine and insulin-induced vasorelaxation) was evaluated in a 5-mL chamber on a Multi Myograph (Danish Myo Technology A/S, Aarhus, Denmark) as previously described (Liu et al. 2009; Sun et al. 2009).

*Histology and immunohistochemistry.* Segments of liver were frozen in liquid nitrogen and embedded in Tissue-Tek OCT compound (Sakura Finetek USA Inc., Torrance, CA, USA) for staining with Oil Red O. Additional paraffinized liver sections were deparaffinized and stained with hematoxylin and eosin (H&E) to observe tissue morphology. In addition, we used immunohistochemistry to identify cell surface glycoprotein F4/80 (F4/80) in liver and VAT sections (Xu et al. 2011).

*Immunoblotting.* VAT and liver were homogenized with M-PER Mammalian Protein Extraction Reagent (Thermo Scientific, Rockford, IL, USA), and proteins were loaded on a 10% SDS-PAGE gel. After electrophoresis, proteins were transferred to Immobilon-P polyvinylidene difluoride membranes (Sigma-Aldrich, St. Louis, MO, USA), which were incubated with different primary antibodies. Antibodies for AKT (protein kinase B) and phosphorylated (P)-AKT (phosphorylation at Ser473), PI3K (phosphatidylinositol 3-kinase), AMPK (AMP-activated protein kinase) and P-AMPK (phosphorylation at Thr172), IRS1 (insulin receptor substrate 1) and P-IRS-1 (phosphorylation at Tyr612), GSK3β (glycogen synthase kinase-3 beta) and P-GSK3β (phosphorylation at Ser9), and MAPK (mitogen-activated protein kinase) pathway proteins were obtained from Cell Signaling Technology (Danvers, MA, USA); and that for PEPCK (phosphoenolpyruvate carboxykinase) was from Santa Cruz Biotechnology (Santa Cruz, CA, USA). After incubation with the primary antibody, the immunoblots were incubated with a horseradish peroxidase–conjugated secondary antibody, visualized with enhanced chemiluminescence, and quantitated by densitometric analysis using ImageJ software (National Institutes of Health, Bethesda, MD, USA). β-Actin was used as a loading control.

*Quantitative reverse-transcriptase polymerase chain reaction (RT-PCR).*RT-PCR was performed using RNA extracted from liver and VAT of mice as described previously (Hagiwara et al. 2012; Xu et al. 2011). Gene expression levels were calculated using the ΔCt method relative to β-actin and are expressed as relative mRNA levels compared with internal control. We used the following primers: *HSL* (hormone sensitive lipase), *ATGL* (adipose triglyceride lipase), *LPL* (lipoprotein lipase), *COX4* (cytochrome c oxidase subunit IV), *COX5A*, *COX7A*, *PGC1*α (peroxisome proliferator-activated receptor gamma coactivator 1α), *PGC1*β, *MCAD* (medium-chain acyl-CoA dehydrogenase), *NrF1* (nuclear respiratory factor 1), *mtTFA* (mitochondrial transcription factor A), *ACO* (acyl-CoA oxidase), *CPT-1* (carnitine palmitoyltransferase 1), *PPAR*α (peroxisome proliferator-activated receptor α), *FABP1* (fatty acid binding protein 1), *FABP2*, *FABP5*, *CD36*, *MTP* (microsomal triglyceride transfer protein), and *APOB* (apolipoprotein B). The sequences of all primers are available upon request.

*Flow cytometric evaluation of inflammation in blood and tissues.* VAT, spleen, and blood from mice (WT-FA, WT-PM, CCR2-FA, and CCR2-PM groups) were processed as described by Kampfrath et al. (2011) and Zhong et al. (2013). Blood cells and spleen cells were incubated with PE-labeled anti-CD11b, FITC-labeled anti-7/4, and PE-Cy7–labeled anti-Gr-1 (Ly-6G/Ly-6C), and the stromal vascular fraction of VAT was incubated with PE-labeled anti-CD11b, PE-Cy5–labeled anti-CD11c (integrin alpha-X), and APC-Cy7–labeled anti-F4/80 (a member of the epidermal growth factor-transmembrane 7 family). All antibodies were purchased from Biolegend (San Diego, CA, USA), Miltenyi Biotec (Bergisch Gladbach, Germany), or BD Biosciences. Cells were then evaluated by flow cytometry using a BD FACS LSR II™ flow cytometer (BD Biosciences), and data were analyzed using BD FACS Diva software (BD Biosciences).

*Electrophoretic mobility shift assay.* Nuclear proteins were extracted from mouse livers using the NE-PER Nuclear and Cytoplasmic Extraction Reagents kit, and the electrophoretic mobility shift assay (EMSA) was conducted using the LightShift kit (both from Pierce, Rockford, IL, USA) according to manufacturer’s instructions. Specificity of the *SREBP1C* (sterol regulatory element-binding protein 1 precursor) probe (5´-GAT CCT GAT CAC CCC ACT GAG GAG-3´) (Seo et al. 2003) was confirmed in assays in which unlabeled *SREBP1c* probe was added in excess as a competitor and by the supershift of *SREBP1c*–DNA complexes.

*Data analysis.* Data are presented as mean ± SE unless otherwise indicated. We used Graphpad Prism software (version 5; GraphPad Software Inc., La Jolla, CA, USA) for one-way analysis of variance (ANOVA) and Bonferroni’s post hoc test where appropriate. When there was no significant difference between WT-PM and WT-FA by one-way ANOVA, we determined exact *p-*values using the *t*-test. We determined EC_50_ values (concentration needed to induce 50% of the maximal effect) using nonlinear regression curve fitting. Concentration-relaxation curves were analyzed by two-way ANOVA followed by Bonferroni’s post-tests. A *p-*value of < 0.05 was considered statistically significant.

## Results

*PM_2.5_ exposure concentration and compositional assessment.* The mean ± SD PM_2.5_ concentrations were 9.56 ± 2.9 μg/m^3^ at the study site (daily ambient level), 2.26 ± 1.9 μg/m^3^ in the FA chamber, and 116.9 ± 34.2 μg/m^3^ in the PM_2.5_ exposure chamber. The concentration in the PM_2.5_ exposure chamber was approximately 12.5 times that in ambient air (see Supplemental Material, Figure S1). The elemental composition of these air samples is available in Supplemental Material, Table S1.

**Figure 1:**
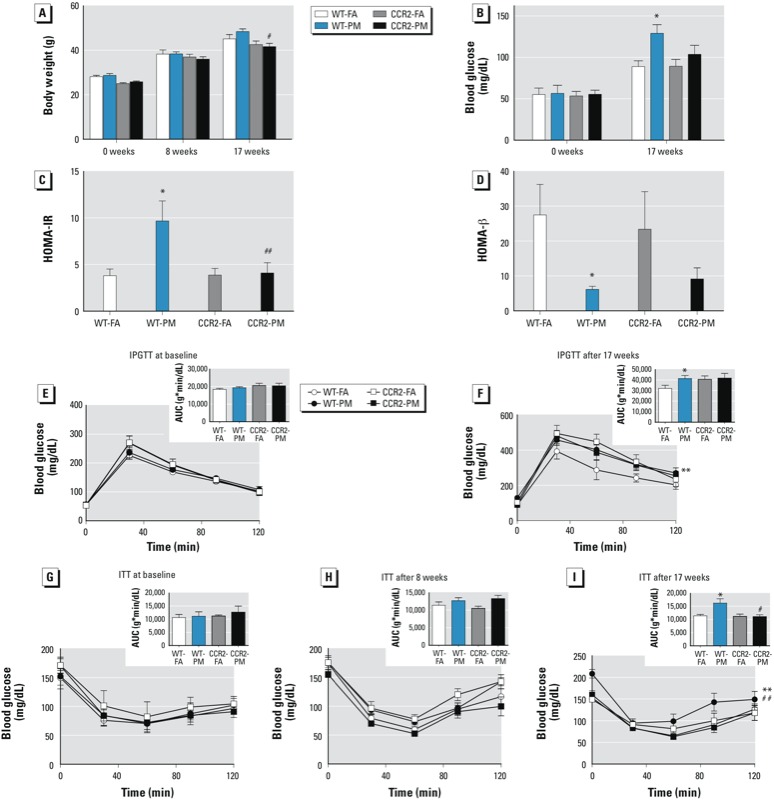
Effect of PM_2.5_ exposure and HFD on glucose homeostasis in WT and CCR2^–/–^ mice; experiments were performed at baseline and after 8 and 17 weeks of exposure to PM_2.5_ or FA. (*A–B*) Body weight (*A*) and fasting blood glucose (*B*). (*C–D*) HOMA-IR (*C*) and HOMA-β (*D*) after 17 weeks of exposure. (*E–F*) Results of IPGTT in overnight-fasted mice at baseline (*E*) and after 17 weeks of exposure (*F*). (*G–I*) Results of ITT in 4.5‑hr–fasted mice before exposure (*G*) and after 8 weeks (*H*) and 17 weeks (*I*) of exposure. Insets (*E–I*) are results of GTT and ITT analyzed by area under the curve (AUC). Values shown are mean ± SE of 7–9 mice/group. *p < 0.05, and **p < 0.01, compared with the WT‑FA group. #*p* < 0.05, and ##*p* < 0.01, compared with the WT‑PM group.

*Role of CCR2 in metabolic impairment by PM_2.5_.* We observed no significant difference between exposure groups in body weight, fasting blood glucose level, glucose tolerance (IPGTT), or insulin sensitivity (ITT) at baseline (prior to consumption of the HFD or assignment to exposure groups) (Figure 1A,B,E,G). After 8 weeks of PM_2.5_ exposure in conjunction with the HFD, we observed no significant difference in body weight (Figure 1A) or insulin sensitivity (Figure 1H) compared with FA-exposed mice. However, at 17 weeks of exposure, the WT-PM group displayed elevated fasting glucose level and HOMA-IR index, decreased HOMA-β function, abnormal glucose tolerance, and attenuation of whole-body insulin sensitivity (Figure 1B–D,F,I). At this time point, CCR2-PM mice had lower body weight and lower blood glucose concentration than did WT-PM mice (Figure 1A,B). CCR2 deficiency did not affect IPGTT values (Figure 1F); however ITT values were statistically significant at 17 weeks (Figure 1I) but not at 8 weeks (Figure 1H). Taken together, these results suggest that CCR2^–/–^ mice are protected from PM_2.5_-induced abnormalities in whole-body insulin sensitivity but that CCR2 is not required for regulation of postprandial glycemic response.

**Figure 2:**
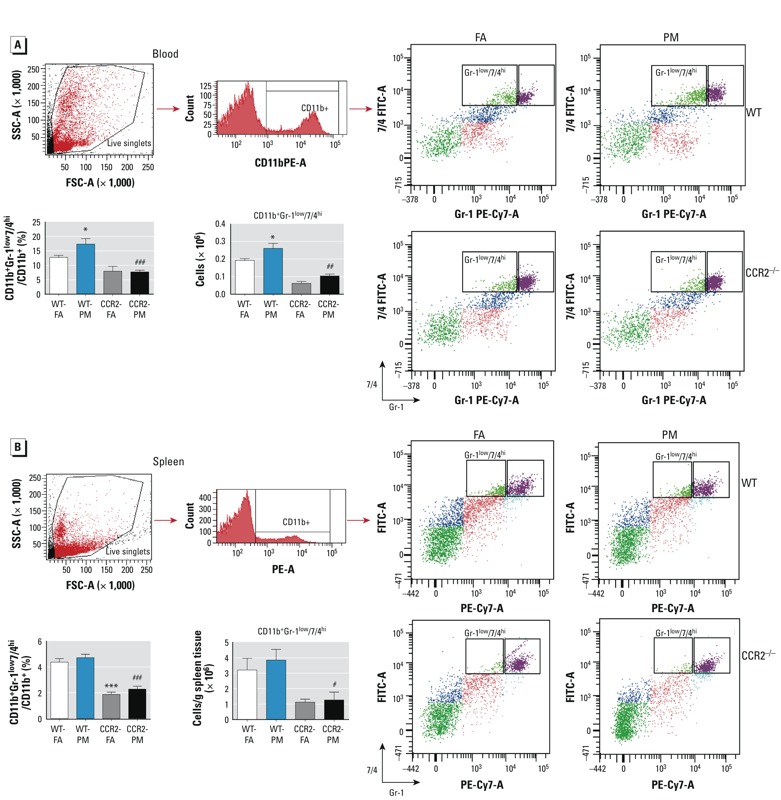
Representative flow cytometric dot plots and analysis showing effects of PM_2.5_ exposure and HFD on inflammatory monocytes in blood (*A*) and spleen (*B*) from WT and CCR2^–/–^ mice; animals were exposed to PM_2.5_ or FA for 17 weeks. Abbreviations: FSC, forward scatter; SSC, side scatter. Data were analyzed by relative percentage or absolute cell counts and are presented as mean ± SE of 7–9 mice/group). *p < 0.05, and ***p < 0.001, compared with the WT‑FA group. #*p* < 0.05, ##*p* < 0.01, and ###*p* < 0.001 for CCR2‑PM compared with the WT‑PM group.

*CCR2 deficiency ameliorates inflammatory monocytes in blood and spleen.* Because CCR2 plays a central role in the egress of monocytes from systemic reservoirs and in depot-specific inflammation (Charo and Ransohoff 2006; Tsou et al. 2007), we investigated the monocyte response to PM_2.5_ exposure. We defined monocytes as side scatter (SSC)–high, forward scatter (FSC)–low cells expressing the myeloid antigen 7/4 (high populations; 7/4^hi^) and high levels of CD11b but low for the neutrophil marker Gr-1 (CD11b^+^Gr-1^low^), which corresponds to Ly6C^hi^ monocytes and represents the inflammatory subtype (Combadiére et al. 2008; Henderson et al. 2003; Kampfrath et al. 2011). We noted an increase in circulating CD11b^+^Gr-1^low^7/4^hi^ cells, the inflammatory subtype in response to PM_2.5_ exposure (Figure 2A). In contrast, splenic levels of CD11b^+^Gr-1^low^7/4^hi^ cells remained unchanged (Figure 2B). The levels of CD11b^+^Gr-1^low^7/4^hi^ in circulation following PM_2.5_ inhalation were significantly reduced in CCR2^–/–^ mice (Figure 2A) with a corresponding decrease in the spleen (Figure 2B). Supplemental Material, Table S2, depicts circulating cytokines in response to PM_2.5_ exposure. The concentration of MCP-1 was significantly higher in CCR2^–/–^ mice than WT mice, whereas there were no differences in other measures.

*CCR2 deficiency does not improve PM_2.5_-impaired endothelium function.* PM_2.5_-exposed C57BL/6 mice demonstrated a decrease in relaxation in response to both acetylcholine and insulin (see Supplemental Material, Figure S2). However, vascular function impaired by PM_2.5_ was not significantly different between CCR2^–/–^ and WT mice (see Supplemental Material, Figure S2). These results suggest that abnormalities in endothelium-dependent relaxation are not modulated through CCR2-dependent pathways in response to air pollution exposure.

**Figure 3:**
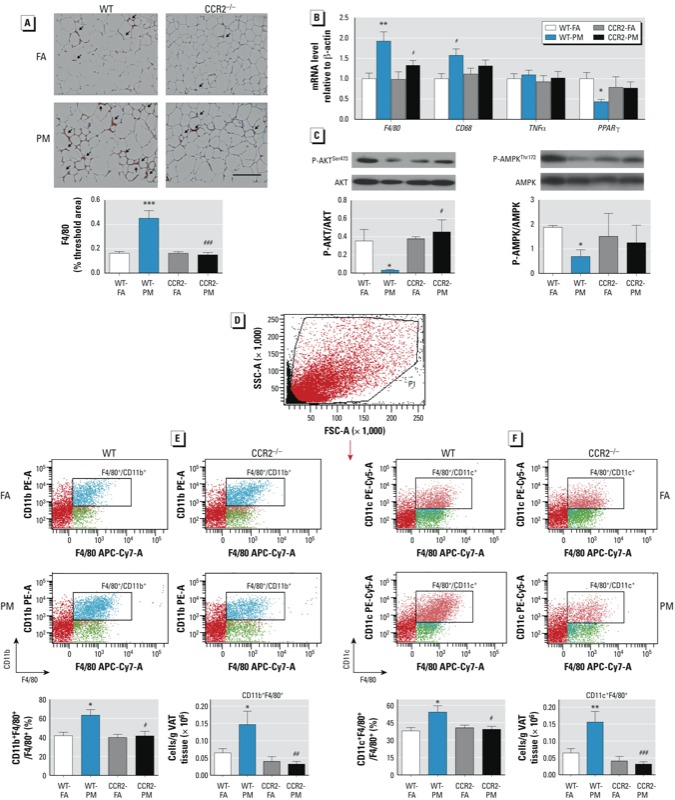
Effect of PM_2.5_ exposure and HFD on inflammation and insulin signaling in VAT from WT and CCR2^–/–^ mice; animals were exposed to PM_2.5_ or FA for 17 weeks. (*A*) Representative image (top; bar = 100 μm) and analysis of F4/80+ staining. (*B*) mRNA levels of genes involved in inflammation (*F4/80*, *CD68*, *TNFα*, and PPARγ). (*C*) Western blots and analysis of phosphorylated AKT (P-AKT)/total AKT (left) and phosphorylated AMPK (P-AMPK)/total AMPK (right). (*D*) Representative flow cytometric dot plot of living cells. (*E–F*) Representative flow cytometric dot plots showing F4/80+/CD11b+ (*E*) and F4/80+/CD11c+ (*F*) with respective analysis by relative percentage or absolute cell count/g VAT. Data are presented as mean ± SE of 7–9 mice/group) *p < 0.05, **p < 0.01, and ***p < 0.001, compared with the WT‑FA group. #*p* < 0.05, ##*p* < 0.01, and ###*p* < 0.001 compared with the WT‑PM group.

*CCR2 modulates adipose inflammation in response to PM_2.5_*. F4/80 is a well-characterized membrane protein that is the best known marker for mature mouse macrophages. In the present study, F4/80^+^ adipose tissue macrophages (ATMs) were increased in VAT of WT-PM mice but not in CCR2-PM mice (Figure 3A). This observation was confirmed by mRNA levels of *F4/80* and an alternate macrophage marker, *CD68* (Figure 3B). PPARγ, a transcription factor required for alternate macrophage differentiation, was down-regulated in VAT of WT-PM mice, but was only partially down-regulated in CCR2-PM mice (Figure 3B). The expression of adipose-derived mediators was not altered by CCR2 deficiency or in response to PM_2.5_ exposure (see Supplemental Material, Figure S3A). As determined by flow cytometry, F4/80^+^/CD11b^+^ and F4/80^+^/CD11c^+^ were increased VAT in response to PM_2.5_ exposure in WT mice but not in CCR2^–/–^ mice (Figure 3D,E).

We found no difference in the weight of VAT between groups (data not shown). We also observed no change in expression of genes involved in lipolysis and mitochondrial oxidation (see Supplemental Material, Figure S3B). NrF1 and mtTFA are transcription factors involved in mitochondrial biogenesis. Neither PM_2.5_ exposure nor CCR2 genotype induced a change in *mtTFA* expression. However, *NrF1* levels were significantly lower in the WT-PM group than that in the WT-FA group, and this was partially restored in CCR2-PM mice (see Supplemental Material, Figure S3B).

**Figure 4:**
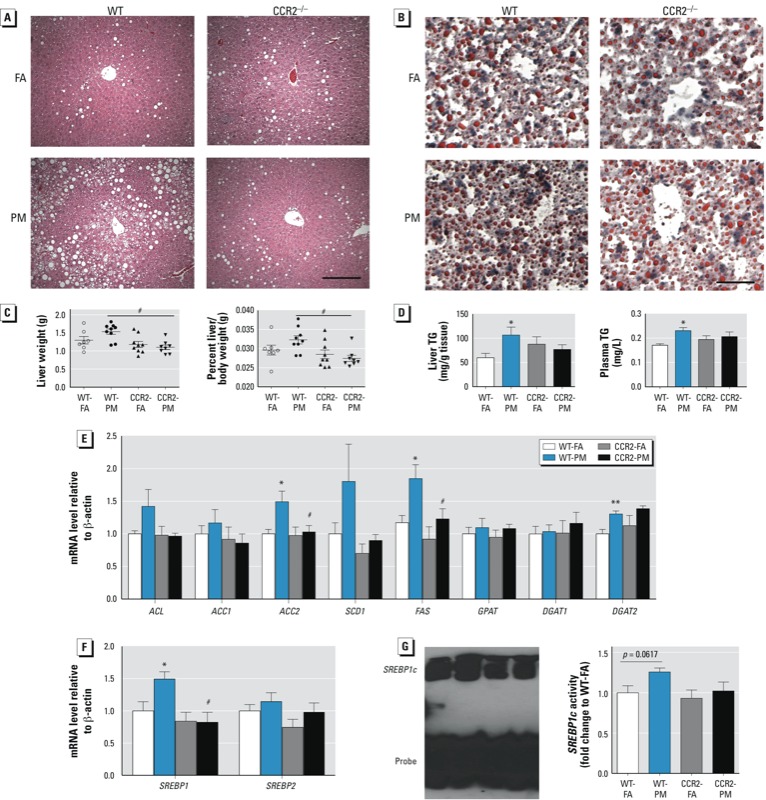
Effect of PM_2.5_ exposure and an HFD on lipid homeostasis in WT and CCR2^–/–^ mice; animals were exposed to PM_2.5_ or FA for 17 weeks. (*A*) Representative images of H&E-stained liver sections; bar = 100 μm. (*B*) Representative images of oil red O–stained liver sections; bar = 25 μm. (*C*) Liver weight (left) and liver weight/body weight ratio (right). (*D*) TG levels in liver (left) and plasma (right). (*E*) mRNA levels of genes involved in de novo lipid synthesis in the liver. (*F*) mRNA levels of SREBP1 and SREBP2. (*G*) The DNA binding activity of SREBP1c in the liver. Data are presented as mean ± SE of 7–9 mice/group. *p < 0.05 for WT‑PM compared with WT‑FA. #*p* < 0.05 for CCR2‑PM compared with the WT‑PM group.

*CCR2 modulates hepatic steatosis in response to PM_2.5_*. Compared with WT-PM mice, CCR2^–/–^ mice showed improved lipid deposition (H&E staining; Figure 4A) and intracytoplasmic lipids (Oil Red O staining; Figure 4B), as well as a trend toward lower liver weight (Figure 4C). In WT-PM mice, levels of hepatic triglycerides and plasma triglycerides were elevated (Figure 4D), suggesting increased production of triglyceride-containing lipoproteins in the liver. We next examined genes involved in lipid metabolism in the liver. Expression of key lipid synthesis enzymes [acetyl-CoA carboxylase 2 (ACC2), fatty acid synthase (FAS), and diacylglycerol acyl transferase (DGAT2)] were all significantly increased in the liver of WT-PM mice compared with WT-FA mice (Figure 4E), whereas there was no difference in expression of other genes. The mRNA level of SREBP1 (a key transcription factor involved in activation of lipogenic genes)—but not SREBP2—was significantly increased in the liver of WT-PM mice (Figure 4F). EMSA of nuclear extracts from the liver demonstrated a trend toward increased SREBP1c binding activity in WT-PM mice, with a smaller increase in CCR2-PM mice (Figure 4G). The increases in lipogenic gene expression observed in WT-PM mice were nearly normal in CCR2-PM mice, with the exception of *DGAT2* (Figure 4E). We observed no significant difference in genes related to fatty acid oxidation (see Supplemental Material, Figure S3C). *FABP1* mRNA—but not *FABP2*, *FABP5,* or *CD36*—was significantly decreased in the liver of WT-PM mice (see Supplemental Material, Figure S3C). Expression of genes encoding fatty acid export, including *APOB* and *MTP* were unaffected by exposure to PM_2.5_ (see Supplemental Material, Figure S3C).

*Role of CCR2 in PM_2.5_-impaired hepatic glucose metabolism.* To investigate mechanisms of hyperglycemia in response to PM_2.5_, we examined pathways involved in gluconeogenesis and glycolysis. We observed no alteration of a rate-limiting enzyme involved in gluconeogenesis, phosphoenolpyruvate carboxykinase (PEPCK), at both mRNA and protein levels (see Supplemental Material, Figure S4A,B). However, we noted inhibition in expression of G6pase, FBPase, and pyruvate carboxylase (PC) in the liver of WT-PM mice compared with that of WT-FA mice (see Supplemental Material, Figure S4A). We found no difference in expression of the transcription factor C/EBP-α, the coactivator (PGC1α), or glycogen synthase kinase 3 beta (GSK3β; regulating glycogen synthase) in the liver of WT-PM animals (see Supplemental Material, Figure S4A,D). These results suggest that enhanced gluconeogenesis or glycogen synthesis is unlikely to contribute to hyperglycemia in response to PM_2.5_ exposure.

We observed no differences in glucokinase (GK), a key glycolytic enzyme, in response to PM_2.5_. However, *GK* expression was increased in the liver of CCR2^–/–^ mice (both FA and PM groups) compared with WT mice (see Supplemental Material, Figure S4C). This may partially explain the reduced glucose levels in CCR2^–/–^ mice. We found a trend of decreased expression of another enzyme of glucose metabolism, L-type pyruvate kinase (*L-PK*). Expression of *GLUT-2* [solute carrier family 2 (facilitated glucose transporter), member 2] was significantly decreased in the liver of WT-PM mice (compared with WT-FA mice), but it was significantly increased in CCR2-PM mice (compared with WT-PM mice). In addition, the transcription factor ChREBP (carbohydrate response element binding protein), indicative of reduced glucose utilization in the liver, was decreased in WT-PM mice. The *ChREBP* level was not modulated by CCR2^–/–^ (see Supplemental Material, Figure S4C). As shown in Supplemental Material, Figure S4E, *GLUT-4* expression in skeletal muscle was decreased in both WT-PM and CCR2-PM mice.

**Figure 5:**
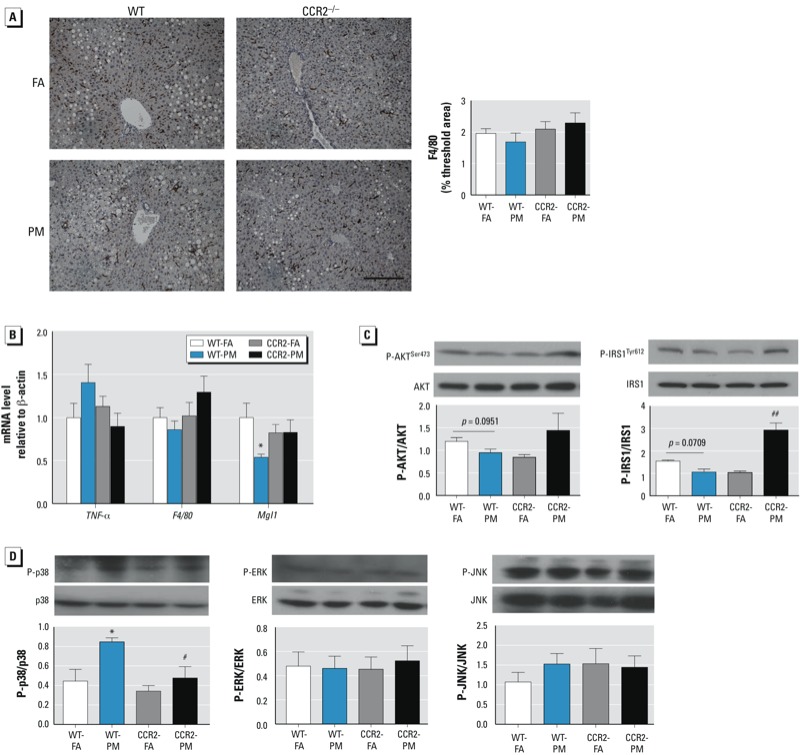
Effects of PM_2.5_ exposure and HFD on inflammation, insulin, and MAPK signaling pathways in the liver of WT and CCR2^–/–^ mice; animals were exposed to PM_2.5_ or FA for 17 weeks. (*A*) Representative image (left; bar = 100 μm) and analysis (right) of F4/80 immuno­staining (*n* = 7–9 mice/group). (*B*) mRNA levels of three genes involved in inflammation: *F4/80*, *TNFα*, and *MgI1* (*n* = 7–9 mice/group). (*C*) Western blot analysis of phosphorylated AKT (P-AKT)/total AKT and phosphorylated IRS1 (P-IRS1)/total IRS1 (*n* = 3–7 mice/group). (*D*) Western blot analysis of signaling molecules involved in the MAPK pathway: phosphorylated p38/p38, phosphorylated ERK/ERK, and phosphorylated JNK/JNK(*n* = 3–5 mice/group). Data are presented as mean ± SE. *p < 0.05, compared with the WT‑FA group. #*p* < 0.05, and ##*p* < 0.01, compared with the WT‑PM group.

*CCR2 modulates hepatic p38 activation in response to PM_2.5_.* To further explore mechanisms by which PM_2.5_ impairs glucose homeostasis and hyperlipidemia, we assessed inflammatory signals implicated in hepatic IR. We noted no differences in F4/80 content in the liver of mice exposed to PM_2.5_, in excess of that induced by HFD (Figure 5A); this was confirmed by quantitative RT-PCR analysis (Figure 5B). The alternative (M2) macrophage activation marker galactose-*N*-acetylgalactosamine-specific lectin (*MgI1*) was down-regulated in WT-PM mice but not in CCR2-PM mice (Figure 5B). Western blot analysis demonstrated that activated p38—but not extracellular signal-regulated kinase (ERK) or c-Jun N-terminal kinase (JNK)—was increased in the liver of PM_2.5_-exposed mice compared with that in FA-exposed mice (Figure 5D). Levels of phosphorylated p38 appeared to be lower in the liver of CCR2^–/–^ mice.

*Defective insulin signaling in VAT and liver.* Phosphorylated AKT (Ser473) was reduced in VAT of WT-PM mice compared with WT-FA mice, but this was not observed in CCR2^–/–^ mice (Figure 3C). Phosphorylated AMPK (Thr172) was inhibited in VAT of WT-PM mice compared with WT-FA mice (Figure 3C), but it was not significantly altered in CCR2-PM mice. Although we observed no significant difference in the liver, there was a clear trend toward a decrease in phosphorylation levels of both AKT at the Ser473 site and insulin receptor substrate-1 (IRS1) at the Tyr612 site in WT-PM mice; however, these levels were increased in the CCR2-PM group (Figure 5C).

## Discussion

In the present study, we delineated the effects of PM_2.5_ inhalation on multiple aspects of glucose/lipid metabolism; our results support a contributing role of CCR2 in PM_2.5_-mediated effects in conjunction with HFD. We found *a*) impairment of systemic insulin sensitivity by PM_2.5_ in WT but not CCR2^–/–^ mice; *b*) CCR2-dependent potentiation of VAT inflammation and impairment of AMPK and AKT signaling by PM_2.5_; *c*) CCR2-dependent enhancement of hepatic lipogenesis/steatosis and activation of p38 MAPK and reduction of insulin signaling by PM_2.5_; and *d*) worsening of fasting hyperglycemia via CCR2-independent nongluconeogenic mechanisms.

We previously reported an important association between PM_2.5_ inhalation and an HFD, providing evidence for an important interaction between environmental and dietary signals (Sun et al. 2009). A component of this effect is recruitment and activation of myeloid cells in tissues such as VAT and liver, where they contribute to adverse metabolic consequences (Oh et al. 2012; Weisberg et al. 2006; Xu et al. 2010; Zheng et al. 2012). Given the importance of the CCR2/CCL2 system in regulating monocyte/macrophage chemotaxis and inflammation in response to HFD signals, we hypothesized that ablation of CCR2 would mitigate adverse consequences of air-pollution exposure in conjunction with an HFD. We did not study normal diet conditions in this investigation because the MCP-1/CCR2 system does not substantially alter inflammation or metabolism in the normal-diet context (Lumeng et al. 2007a; Obstfeld et al. 2010; Weisberg et al. 2006).

PM_2.5_ exposure attenuated whole-body insulin sensitivity and glucose homeostasis after a substantial latency period (> 8 weeks). In keeping with our original hypothesis, we noted increased numbers of immune cells in the peripheral circulation and VAT in response to PM_2.5_ exposure, which was not present in CCR2^–/–^ mice, suggesting a dependence of PM_2.5_ on CCR2 in recruitment of innate immune cells (Ito et al. 2008; Tsou et al. 2007; Weisberg et al. 2006). Infiltration of monocytes is enhanced in obesity via local tissue cues, with a progressive transformation of these cells to a CD11c^+^ status, resulting in a polarization of the local adipose milieu to an M1 state from a predominantly M2 state under conditions of normal diet (Lumeng et al. 2007b; Oh et al. 2012). Given the significantly higher numbers of CD11c^+^ cells (absolute numbers) in WT-PM_2.5_ mice, our results suggest that these cells in VAT may be a consequence of recruitment rather than polarization of existing cell populations.

A key defect in IR is abnormal insulin signaling through alterations in the IRS1–PI3K–AKT pathway. The reduced phosphorylation of the downstream signaling mediator AKT is well implicated as a key marker of IR and has been strongly linked to inflammatory triggers in VAT (Lumeng et al. 2007a, 2007b; McGillicuddy et al. 2009; Osborn and Olefsky 2012; Sun et al. 2009). Similarly, abnormalities in AMP-kinase signaling have been noted as a potential target of inflammation in metabolic diseases (Canto et al. 2009; Salminen et al. 2011; Yu et al. 2010). Reduction in phosphorylated AKT and AMPK in VAT in response to PM_2.5_ exposure in WT mice—with no reduction in CCR2^–/–^ mice—suggests a dependence of abnormal signaling on inflammation in these pathways. Similarly, in livers from the WT-PM group, we noted a clear trend toward a decrease in levels of phosphorylated AKT and phosphorylated IRS1 at Tyr 612, which was not observed in the CCR2-PM group. These results complement our prior work, which clearly demonstrated increased Ser 636 and Ser 1101 phosphorylation in the liver from mice exposed to PM_2.5_, collectively suggesting a PM_2.5_-triggered inhibition of IRS1 signaling (Zheng et al. 2012).

Obesity is well known to induce hepatic triglyceride accumulation and fatty liver, a process coordinated by broad transcriptional programs governing carbohydrate and lipid metabolism. CCR2/CCL2 has previously been shown to regulate triglyceride accumulation (Baeck et al. 2012; Mandrekar et al. 2011). We found that triglyceride levels and neutral fat deposition were markedly higher in WT-PM mice compared with the WT-FA group, which may partly explain the increased liver mass. SREBP1c activation in response to PM_2.5_ exposure is likely critical to the up-regulation of multiple enzymes involved in triglyceride synthesis. We noticed FABP1, a protein highly expressed in tissues (i.e., liver) that is active in long-chain fatty acid uptake and metabolism, was down-regulated in response to PM_2.5_ exposure in WT mice but not in CCR2^–/–^ mice. Martin et al. (2008) reported that FABP1-ablated mice exhibited increased age-dependent obesity, which is in line with our study. Taken together, increased lipogenesis and decreased fatty acid uptake, but not fatty acid oxidation or lipid export pathways, account for excess triglyceride accumulation in the liver in response to PM_2.5_ exposure.

p38 MAPK belongs to a family of evolutionarily conserved serine–threonine MAPKs that link extracellular signals to intracellular machinery regulating a plethora of cellular processes. Together with JNK, they are activated by environmental or genotoxic stress and described as stress-activated protein kinases (Chang and Karin 2001; Coulthard et al. 2009; Morrison and Davis 2003) Consistent with studies that demonstrated the role of p38 in mediating adverse consequences (Liu and Cao 2009), in the present study, we found that p38 was selectively up-regulated in response to PM_2.5_, with the effect stronger in WT than in CCR2^–/–^ mice. However, Lee et al. (2011) have suggested a protective effect in which increases in p38 activity may regulate Xbp1 nuclear translocation and activity, and thus may represent a compensatory mechanism to maintain homeostatic response. Therefore, the significance of this finding may need additional study.

Circulating glucose levels reflect a balance between glucose production and utilization. Skeletal muscle, which accounts for approximately 80% of insulin-stimulated whole-body glucose disposal, is by far the most affected organ with respect to impaired insulin-stimulated glucose disposal in states of IR. GLUT-4 expression in skeletal muscle was decreased in response to PM_2.5_ exposure in WT mice, indicating a defect in glucose utilization. Interestingly, a decrease in GLUT-4 levels also occurred in CCR2^–/–^ mice and may represent a potential explanation for lack of improvement in glucose-tolerance. Gluconeogenesis is tightly regulated by insulin signaling (suppressed), with mitigation of this suppression with IR (in the face of continued insulin-mediated lipogenesis). This process requires coordinated activity of four enzymes: PEPCK, G6pase, FBPase, and PC (Jitrapakdee 2012). Surprisingly, we found reduced expression of G6pase, FBPase, and *PC* mRNA levels, with no alteration of *PEPCK* levels, after PM_2.5_ exposure, suggesting an adaptive negative feedback regulation of gluconeogenesis. We found no difference in expression of transcription factors responsible for regulating gluconeogenesis/​glycogen synthesis in liver of WT-PM animals, suggesting that enhanced gluconeogenesis or glycogen synthesis is unlikely to contribute to hyperglycemia in response to PM_2.5_ exposure. Using the DNA motif of the *L-PK* gene as an affinity tag, Uyeda and Repa (2006) purified a transcription factor from nuclear extracts of liver tissue, which was named ChREBP. Decreased ChREBP in response to PM_2.5_ exposure may provide an explanation for a trend of glycolysis inhibition. In contrast, GLUT-2, a transporter in liver cells that functions to mediate glucose uptake in the liver for glycolysis, was reduced by PM_2.5_ exposure. This may contribute to attenuated glucose uptake in the liver and PM_2.5_-mediated hyperglycemia in the present study. Although CCR2^–/–^ mice showed no improvement in *ChREBP* or *L-PK*, the normalized *GLUT-2* expression and *GK* overexpression in these mice may be expected to alleviate glucose dysregulation induced by PM_2.5_ exposure. Additional experimentation will be required to clarify the mechanism.

In summary, the present study demonstrates complex effects of PM_2.5_ in exaggerating effects of an HFD. CCR2 plays important roles in adverse effects of PM_2.5_ by modulating VAT inflammation and hepatic steatosis but not glucose utilization in skeletal muscle. These findings provide new mechanistic links between air pollution and metabolic abnormalities.

## Supplemental Material

(946 KB) PDF
