# Supplementary material for: Air Pollution–Mediated Susceptibility to Inflammation and Insulin Resistance: Influence of CCR2 Pathways in Mice
Source: Environ Health Perspect. 2013 Oct 22;122(1):17–26. doi: 10.1289/ehp.1306841 (PMC3888572; doi:10.1289/ehp.1306841)
Supplement: (946 KB) PDF [file ehp.1306841.s001.508.pdf]

*Environmental Health Perspectives* doi:10.1289/ehp.1306841

# Supplemental Material
# Air Pollution–Mediated Susceptibility to Inflammation and Insulin Resistance: Influence of CCR2 Pathways in Mice

Cuiqing Liu, Xiaohua Xu, Yuntao Bai, Tse-Yao Wang, Xiaoquan Rao, Aixia Wang, Lixian Sun, Zhekang Ying, Liubov Gushchina, Andrei Maiseyeu, Masako Morishita, Qinghua Sun, Jack R. Harkema, and Sanjay Rajagopalan

**Table of Contents**


| **Content** | **Pages** |
|---|---|
| Supplemental Material, Table S1 | 2 |
| Supplemental Material, Table S2 | 3 |
| Supplemental Material, Figure S1 | 4 |
| Supplemental Material, Figure S2 | 5 |
| Supplemental Material, Figure S3 | 6-7 |
| Supplemental Material, Figure S4 | 8 |

Supplemental Material, Table S1. Elemental constituents of air from OASIS in Columbus, Ohio, December 2011 to March 2012 by energy-dispersive X-ray fluorescence.

| **Elements** | **Ambient Air** | **Filtered Air** | **$PM_{2.5}$ Air** |
|---|---|---|---|
| S | 672.0 ± 146.6 | -0.1 ± 43.6 | 6020.0 ± 3292.9 |
| Ca | 74.0 ± 22.1 | 66.2 ± 40.5 | 545.7 ± 304.5 |
| Na | 72.6 ± 34.2 | 47.0 ± 29.8 | 412.4 ± 247.5 |
| Fe | 42.0 ± 21.0 | 21.1 ± 23.2 | 353.8 ± 219.1 |
| K | 39.3 ± 15.0 | 22.8 ± 18.0 | 263.8 ± 159.1 |
| Zn | 21.1 ± 15.5 | 7.8 ± 12.5 | 182.7 ± 180.1 |
| Mg | 19.9 ± 7.8 | 11.2 ± 5.2 | 144.9 ± 79.6 |
| Al | 16.1 ± 10.1 | 18.4 ± 22.6 | 142.5 ± 126.9 |
| P | 12.8 ± 8.9 | 14.0 ± 5.4 | 86.3 ± 93.6 |
| Pb | 3.2 ± 1.4 | 0.3 ± 0.9 | 23.8 ± 15.5 |
| Cu | 2.2 ± 1.1 | 1.5 ± 2.2 | 17.8 ± 10.4 |
| Ba | 2.2 ± 0.7 | 0.6 ± 0.6 | 17.6 ± 9.8 |
| Mn | 1.8 ± 1.0 | 0.5 ± 0.3 | 16.0 ± 11.2 |
| Cr | 2.2 ± 0.5 | 5.0 ± 1.9 | 6.4 ± 3.0 |
| Se | 0.6 ± 0.2 | 0.0 ± 0.0 | 5.8 ± 3.4 |
| Ti | 0.6 ± 0.2 | 0.1 ± 0.1 | 5.2 ± 2.9 |
| Sb | 0.6 ± 0.1 | 0.0 ± 0.0 | 4.4 ± 2.4 |
| Sr | 0.4 ± 0.2 | 0.2 ± 0.1 | 3.6 ± 2.2 |
| *As* | *0.4 ± 0.1* | *0.0 ± 0.0* | *3.4 ± 1.9* |
| Mo | 0.4 ± 0.2 | 0.2 ± 0.3 | 2.8 ± 1.5 |
| Ni | 0.2 ± 0.3 | 0.1 ± 0.5 | 1.8 ± 1.5 |
| V | 0.2 ± 0.1 | 0.0 ± 0.0 | 1.4 ± 0.9 |
| Cd | 0.1 ± 0.1 | 0.1 ± 0.1 | 0.9 ± 0.5 |
| Rb | 0.1 ± 0.0 | 0.0 ± 0.0 | 0.6 ± 0.4 |
| Ce | 0.0 ± 0.0 | 0.0 ± 0.0 | 0.4 ± 0.3 |
| La | 0.0 ± 0.0 | 0.0 ± 0.0 | 0.3 ± 0.2 |
| Co | 0.0 ± 0.0 | 0.1 ± 0.0 | 0.2 ± 0.1 |

Units are ng/mg; $n$ = 14 filters. Data are means ± SD.

Supplemental Material, Table S2. Effect of $PM_{2.5}$ exposure on circulating inflammatory cytokines in WT and CCR2-/- mice fed an HFD.

| | **Groups** | | | |
|---|---|---|---|---|
| **Items** | **WT-FA** | **WT-PM** | **CCR2-FA** | **CCR2-PM** |
| TNFα (pg/ml) | 9.7 ± 0.6 | 15.6 ± 3.0 | 12.4 ± 2.9 | 11.4 ± 1.4 |
| IL-6 (pg/ml) | 5.1 ± 0.4 | 11.2 ± 5.1 | 5.3 ± 0.5 | 9.0 ± 1.8 |
| MCP-1 (pg/ml) | 32.0 ± 2.2 | 33.0 ± 2.2 | 111.2 ± 21.1*** | 120.4 ± 14.7***$^{###}$ |
| IFNγ (pg/ml) | 1.8 ± 0.2 | 2.4 ± 0.8 | 2.1 ± 0.4 | 1.8 ± 0.2 |
| IL-12 p70 (pg/ml) | 10.8 ± 1.3 | 8.5 ± 2.2 | 15.1 ± 4.0 | 8.4 ± 0.9 |

Note: ***$p < 0.001$ compared with WT-FA group, ###$p < 0.001$ compared with WT-PM group. Data are expressed as means ± SEM. $n$ = 7-9 per group.

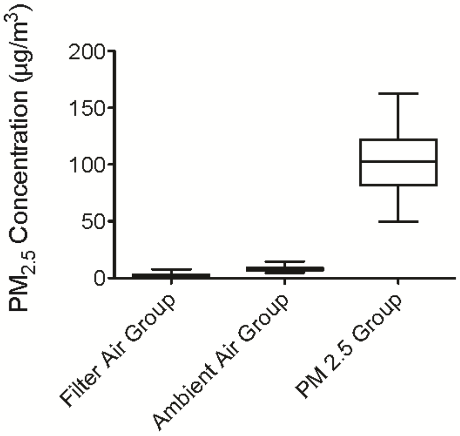


Supplemental Material, Figure S1. $PM_{2.5}$ concentration to which mice were exposed at the study site. Data are means ± SD of 9-12 filters.

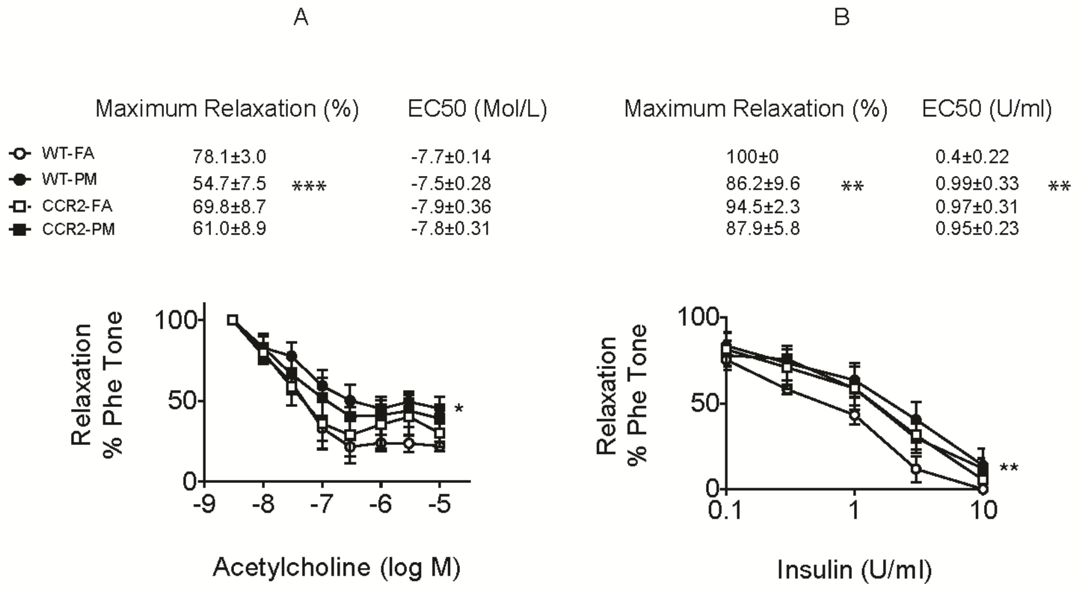


Supplemental Material, Figure S2. Effect of $PM_{2.5}$ exposure on endothelium-dependent vascular relaxation in aorta from HFD-fed mice. A-B, Maximum relaxation, $EC_{50}$ and dose-response to acetylcholine (A) and insulin (B) in aortic rings precontracted with phenylephrine. *$p < 0.05$, **$p < 0.01$, ***$p < 0.001$ when WT-PM compared to WT-FA group. $n$ = 7-9 per group.

A

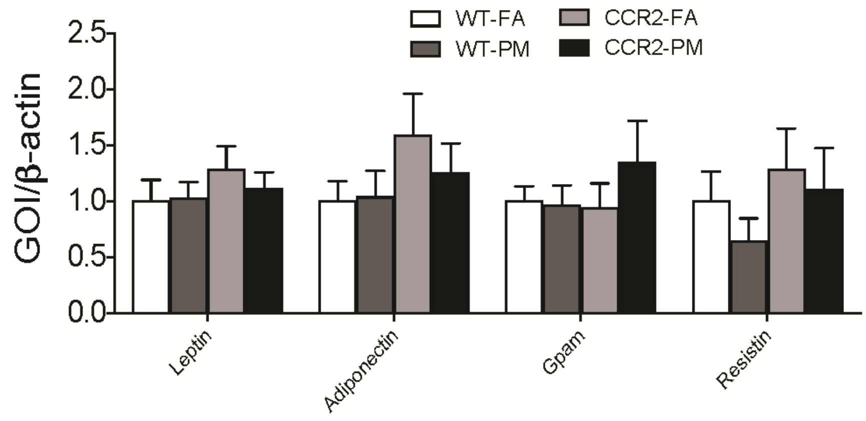


B

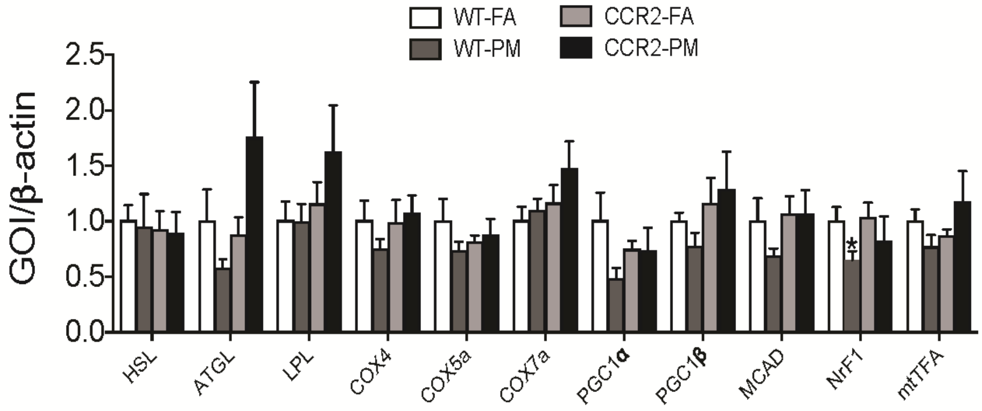


C

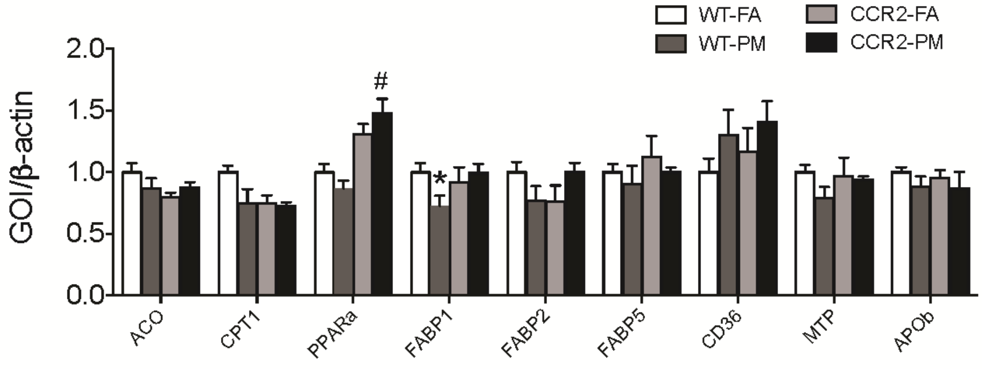


Supplemental Material, Figure S3. Effect of $PM_{2.5}$ exposure on mRNA levels of genes in VAT and liver of HFD-fed mice. A, mRNA levels of genes involved in adipocyte function in VAT. B, mRNA levels of genes involved in lipolysis and mitochondrial oxidation and/or biogenesis in VAT. Hormone sensitive lipase (HSL), Adipose triglyceride lipase (ATGL), Lipoprotein lipase

(LPL) are involved in lipolysis. Cytochrome c oxidase subunit VI (COX4), Va (COX5a), VIIa (COX7a), peroxisome proliferator-activated receptor gamma coactivator 1 α and β (PGC1α, PGC1β), and medium-chain acyl-CoA dehydrogenase (MCAD) are involved in mitochondrial oxidation. Nuclear respiratory factor 1 (NRF1) and mitochondrial transcription factor A (mtTFA) are involved in mitochondrial biogenesis. C, mRNA levels of genes involved in lipid metabolism in the liver. Acyl-CoA oxidase (ACO), Carnitine palmitoyltransferase 1 (CPT-1), and PPARα are involved in β-oxidation. Fatty acid binding protein 1 (FABP1), FABP2, FABP5, and CD36 are involved in fatty acid uptake. Microsomal triglyceride transfer protein (MTP) and Apolipoprotein b (Apob) are involved in VLDL secretion.*$p < 0.05$ when WT-PM compared to WT-FA group, #$p < 0.05$ when CCR2-PM compared to WT-PM group. $n = 7\text{-}9$ per group.

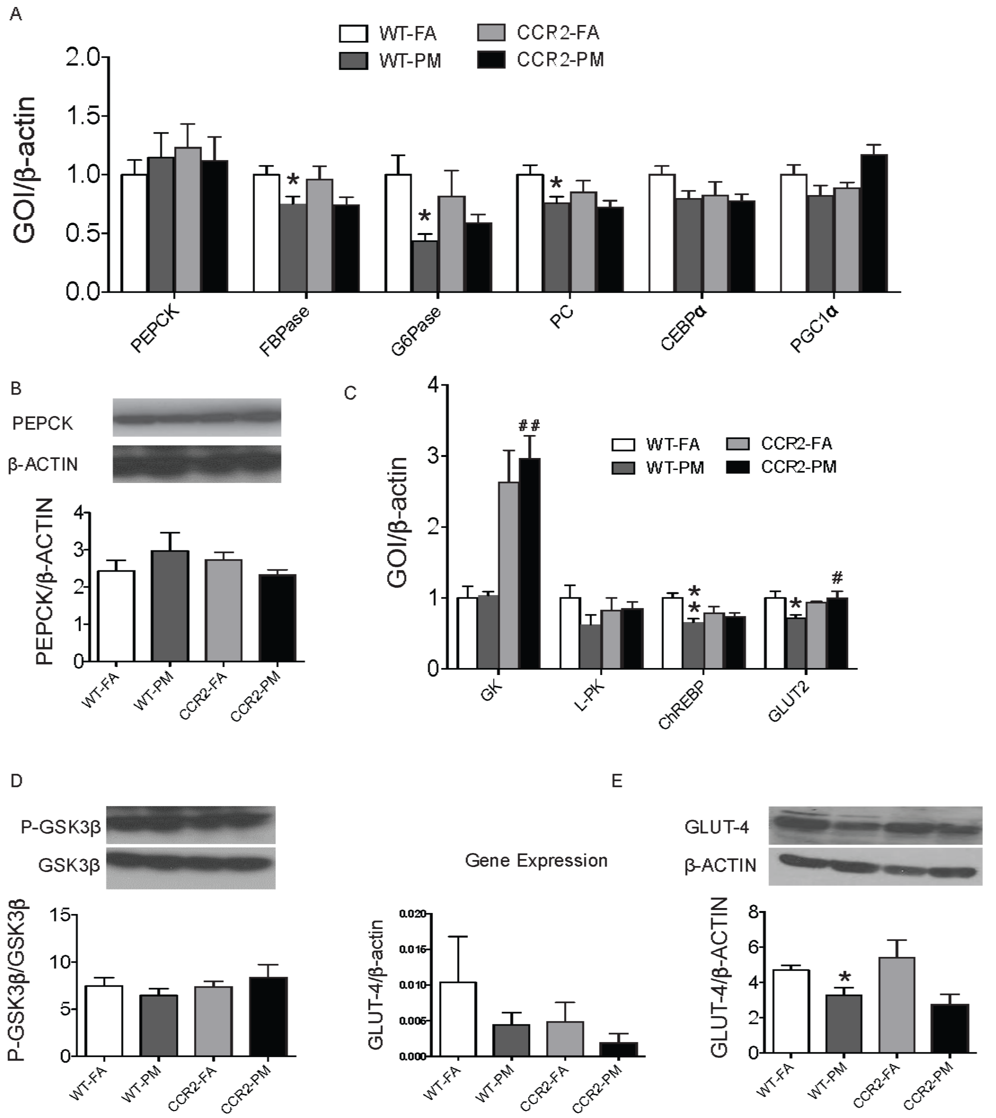


Supplemental Material, Figure S4. Effect of $PM_{2.5}$ exposure on glucose metabolism-related signals in the liver and muscle of HFD-fed mice. A, mRNA levels of gluconeogenesis-related genes in the liver. B and D, Western blotting for PEPCK (B) and phospho-GSK3β/total GSK3β (D) in the liver. C, mRNA levels of glycolysis-related gene in the liver. E, mRNA and protein levels of GLUT-4 expression in the skeletal muscle. *$p < 0.05$ when WT-PM compared to WT-FA group, #$p < 0.05$, ## $p < 0.01$ when CCR2-PM compared to WT-PM group. $n$ = 5-9 per group.
